# Performance of a novel computer‐aided diagnosis system in the characterization of colorectal polyps, and its role in meeting Preservation and Incorporation of Valuable Endoscopic Innovations standards set by the American Society of Gastrointestinal Endoscopy

**DOI:** 10.1002/deo2.178

**Published:** 2022-10-28

**Authors:** Ejaz Hossain, Mohamed Abdelrahim, Andrea Tanasescu, Masayoshi Yamada, Hiroko Kondo, Shijemi Yamada, Ryuji Hamamoto, Atsushi Marugame, Yutaka Saito, Pradeep Bhandari

**Affiliations:** ^1^ Portsmouth Hospitals NHS Trust Portsmouth UK; ^2^ National Cancer Center Hospital Endoscopy Division Tokyo Japan; ^3^ National Cancer Center Research Institute Division of Molecular Modification and Cancer Biology Tokyo Japan; ^4^ RIKEN Center for Advanced Intelligence Project Tokyo Japan; ^5^ NEC Corporation Medical AI Research Department Tokyo Japan; ^6^ University of Portsmouth, University House Portsmouth UK

**Keywords:** artificial intelligence, cancer, characterization, colorectal, polyps

## Abstract

**Background and aims:**

There has been an increasing role of artificial intelligence (AI) in the characterization of colorectal polyps. Recently, a novel AI algorithm for the characterization of polyps was developed by NEC Corporation (Japan). The aim of our study is to perform an external validation of this algorithm.

**Methods:**

The study was a video‐based evaluation of the computer‐aided diagnosis (CADx) system. Patients undergoing colonoscopy were recruited to record videos of colonic polyps. The frozen polyp images extracted from these videos were used for real‐time histological prediction by the endoscopists and by the CADx system, and the results were compared.

**Results:**

A total of 115 polyp images were extracted from 66 patients. Sensitivity, negative predictive value and accuracy for diminutive polyps on white light imaging (WLI) and image‐enhanced endoscopy (IEE) when assessed by CADx was 90.9% [95% confidence interval (CI) 77.3–100] and 95.8% [95% CI 87.5–100], 80% [95% CI 44.4–97.5] and 90.9% [95% CI 58.7–99.8], 84.8% [95% CI 72.7‐97] and 84.6% [95%CI 71.8‐94.9], respectively, compared to 48.1% [95%CI 37.7–59.1] and 72% [95% CI 62.5–81], 37.5% [95% CI 28.8–46.8] and 55% [95% CI 44.7–65.0], 53.7% [95% CI 44.2–63.2] and 66.7% [95% CI 59.7–73.3] when assessed by endoscopists. Concordance between histology and CADx‐based post‐polypectomy surveillance intervals was 93.02% on WLI and 96% on IEE.

**Conclusion:**

AI‐based optical diagnosis is promising and has the potential to be better than the performance of general endoscopists. We believe that AI can help make real‐time optical diagnoses of polyps meeting the Preservation and Incorporation of Valuable endoscopic Innovations standards set by the American Society of Gastrointestinal Endoscopy.

## INTRODUCTION

Detection and removal of adenomas during colonoscopy have been shown to reduce the risk of colorectal cancer.^1^, ^2^, ^3^, ^4^ However, most polyps found during colonoscopy are diminutive (1–5 mm) and the risk of cancer in them is almost negligible.^5^, ^6^, ^7^, ^8^, ^9^, ^10^, ^11^ Removing these polyps and sending them for pathological evaluation can be associated with significant cost, time, and adverse events.

Distal hyperplastic polyps harbor no malignant potential and therefore if recognized accurately, can safely be left in situ (“diagnose and leave”). Similarly, diminutive adenomas anywhere in the colon can be resected and not retrieved for histopathology (“resect and discard”). The American Society of Gastrointestinal Endoscopy has set Preservation and Incorporation of Valuable endoscopic Innovations (PIVI) standards for use of in‐vivo diagnosis in place of conventional histology to practice strategies like “diagnose and leave” and “resect and discard”.^12^, ^13^, ^14^

A lot of studies have shown that image enhancement technologies in expert hands can meet the PIVI criteria.^15^, ^16^, ^17^, ^18^ This has led to various calls for the implementation of this technique in clinical practice. However, attempts at a more widespread generalization of optical diagnosis have failed due to the inability of general endoscopists to meet the established standards.^15^, ^19^, ^20^ As a result, the current practice still requires all polyps to be both resected and retrieved for histological assessment by pathologists.

In recent years, deep learning has evolved and allowed the development of AI algorithms that can characterize polyps in real time without the need for special endoscopes or special training. However, most of the work done for polyp characterization, so far, has been with patented image enhancement technologies like narrow‐band imaging (NBI),^21^, ^22^ blue light imaging (BLI)^23^ or chromoendoscopy, and the AI platforms developed are vendor‐based working on single patented technologies.

Recently, a novel AI algorithm for the characterization of polyps was developed by NEC Corporation (Japan).^24^ The unique feature of this algorithm is that it is not vendor specific and also works on white light imaging (WLI). However, the algorithm was developed using videos from a single Japanese center (NCCH, Tokyo. Japan).

The aim of our study is to perform an external validation of this algorithm with polyp videos collected from two different endoscopy platforms (Olympus and Fujifilm) and also to compare the performance of the AI system against general endoscopists from the west.

## METHODS

### Study design

This study was conducted using prospectively collected endoscopy videos. Assessments for AI and endoscopists were done using corrected videos. Therefore, there is no intervention in patient care. Sixty‐six consecutive adult patients undergoing colonoscopy at Portsmouth Hospitals NHS Trust were recruited into the study. Patients with polyposis syndromes, inflammatory bowel disease, or those unable to give consent were excluded.

The study was a video‐based evaluation of the CADx characterization system. The study was approved by the central ethics committee and was registered with clinicaltrials.gov (NCT04937647). Patients undergoing colonoscopy were recruited to record videos of colonic polyps with and without image enhancement. The endoscopes and processors used for colonoscopy were from Olympus and Fujifilm.

**FIGURE 1:**
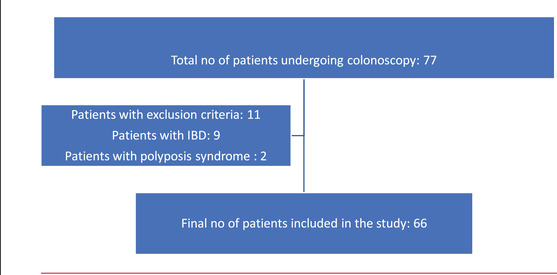
Flowchart depicting enrolment of selected cases

Endoscopists recorded the procedures with freeze shots of polyps on WLI and IEE as well as the polyp morphology, location, and size. The frozen Polyp images were extracted from these videos as PNGs and used for real‐time histological prediction by the Endoscopists and by the CADx system (WISE VISION; NEC Corporation). All detected polyps were removed and sent for histological assessment by expert GI Pathologists. The histological report formed the gold standard against which the performance of CADx and optical diagnosis of endoscopists was compared. Figure 1 demonstrates the enrollment of the selected cases.

### Statistics and sample size estimation

This is a superiority trial aimed to demonstrate the superiority of the CADx system over the Endoscopists for histology prediction. Sample sizes were calculated for WLI and IEE separately. Previously published data^14^ define 80% sensitivity and 80% specificity as the competence standards to implement the resect‐and‐discard strategy as well as estimate accuracy. The accuracy of the AI obtained in the pilot study was equivalent to almost 80%. Based on the above data, we decided to conduct a validation study to test whether AI is clinically acceptable.

We assumed the accuracy of AI to be 77.9% as compared to 59.3% for the endoscopists on WLI and 85.7% accuracy of AI compared to 70.8% for endoscopists on IEE. Accounting for a misalignment exclusion rate of 0.6% for WLI and 7.5% for IEE noted in the pilot study, we calculated the sample size for 80% power assuming a 5% significance level. This meant that we would require at least 53 polyps with WLI and another 53 on IEE if CADx had to be compared with seven independent endoscopists. *p*‐alues were calculated using the Bootstrap method.

### Endoscopists

A total of seven endoscopists from Portsmouth Hospitals NHS Trust were selected for the study. All were independent accredited practitioners with a range of experience, from 500–2000 colonoscopies. A dedicated training session on optical diagnosis was conducted for all the trial endoscopists where the NICE(NBI) and BASIC(BLI) classifications were taught, an image library was reviewed and a structured assessment was performed to ensure that they had a good grasp of the classifications.

The test images were randomly presented to all participants to make an optical diagnosis of polyps into two categories: ‘neoplastic’ for adenomatous polyps and ‘non‐neoplastic’ for non‐adenomatous polyps. They were also asked to state their degree of confidence (high or low) in the optical diagnoses made by them.

### CADx system (WISE VISION; NEC Corporation)

The system was developed by NEC Corporation, Japan in collaboration with National Cancer Centre Hospital, Tokyo.

### Development and internal validation of the AI system

The deep learning model, based on ResNet152 architecture, was developed using 55,890 images (over 11,068 lesions) of histologically‐proven colorectal polyps provided by NCCH, Tokyo. Data was split in an 80:20 ratio into independent training and validation sets. Annotation of polyps by trained endoscopists and histology formed the ground truth for detection and characterization, respectively.

On the internal validation set (8557 polyp images), the model achieved an overall accuracy of 92.8%. The sensitivity for serrated lesions (which includes hyperplastic as well as sessile serrated lesions), adenoma, carcinoma, and normal mucosa predictions were 87.1%, 83.3%, 85.8%, and 100%, respectively. Specificity was 98.0%, 97.5%, 96.3% and 99.5%, respectively.

### Design of the AI system

WISE VISION is designed to analyze video sequences in real time to detect colorectal polyps and classify them as either low possibility (includes hyperplastic and sessile serrated lesion) or high possibility (includes adenoma and cancer). The system has been designed to be easily connected to all major endoscopy processors and uses a separate monitor to display its detection and characterization in real time. Detection is signaled with a bounding box and a sound alarm, while characterization is displayed on the side of the screen when a still image of the polyp is taken. The system can predict histopathological diagnosis at 20.4 ms/image on average using one GPU.

## RESULTS

### Study images and lesion characteristics

A total of 115 images from 115 polyps were extracted from 66 consecutive patients undergoing colonoscopy. None of the images used magnification or near focus. A total of 52 images with WLI and 63 images with IEE were collected using Olympus (Evis X1) and Fujifilm (Eluxeo 7000) processors. Of which, 37/52 (71.1%) WL images and 43/63 (68.3%) IEE images had neoplastic polyps and the rest were non‐neoplastic. The histological distribution of the selected polyps was as follows: hyperplastic (27), sessile serrated lesion (8), and adenoma (80). There were no cancerous lesions in the study. Seventy‐two polyps were ≤ 5 mm in size and 43 polyps were > 5 mm in size. A total of 26 polyps ≤5 mm in size were found in the recto‐sigmoid. The mean size of polyps included in the study was 5.1 mm. (range 5–13 mm). The core characteristics of the polyps have been summarized in Table 1.

**TABLE 1:** Lesion characteristics

	**Hyperplastic**	**Adenoma**	**SSL**
≤ 5 mm (*N* = 72)	20	46	6
>5 mm (*N* = 43)	7	34	2

Abbreviation: SSL, sessile serrated lesion.

### Performance of CADx versus endoscopists on WLI

On WLI, the overall sensitivity of AI for recognition of neoplasia was significantly better than that of the endoscopists (91.9% vs. 55.2%, *p* < 0.001). We found that the overall negative predictive value (NPV) for AI was 75% compared to 34.7% (*p* = 0.01) for endoscopists. On sub‐group analysis for polyps ≤5 mm, the NPV for AI was significantly better at 80% compared to 37.5% for endoscopists (*p* = 0.015). Similarly, the overall accuracy for polyps ≤5 mm, AI was significantly superior to the endoscopists but despite a numerical trend statistical significance was not achieved for polyps >5 mm (Table 2).

**TABLE 2:** Computer‐aided diagnosis (CADx) versus endoscopists on white light imaging

Polyp size	Evaluator	Sensitivity (95% CI)	*p*‐value	Specificity (95% CI)	*p*‐value	NPV (95% CI)	*p*‐value	Accuracy (95% CI)	*p*‐value
≤5 mm	AI	90.9 (77.3–100)	<0.001	72.7 (45.5–100)	0.651	80.0 (44.4–97.5)	0.015	84.8 (72.7–97)	<0.001
	Endoscopist	48.1 (37.7–59.1)		64.9 (48.1–80.5)		37.5 (28.8–46.8)		53.7 (44.2–63.2)	
>5 mm	AI	93.3 (80.0–100)	0.015	25.0 (0.0–75)	1.000	50.0 (1.3–98.7)	0.494	78.9 (57.9–94.7)	0.155
	Endoscopist	65.7 (52.4–78.1)		53.6 (14.3–92.9)		27.7 (15.6–42.6)		63.2 (49.6–75.9)	
Overall	AI	91.9 (81.1–100)	<0.001	60.0 (33.3–86.7)	1.000	75.0 (42.8–94.5)	0.01	82.7(71.2–92.3)	<0.001
	Endoscopist	55.2 (46.7–64.1)		61.9 (44.8–77.1)		34.7 (27.5–42.5)		57.1 (49.7–64.8)	

Abbreviations: AI, artificial intelligence; NPV, negative predictive value.

### Performance of CADx versus endoscopists on IEE

On IEE using BLI and NBI, the overall sensitivity for AI in the recognition of neoplasia was significantly better than that of the endoscopists (95.3 vs. 76.4, *p* < 0.001)

We found that the overall NPV for AI was significantly better at 86.7% compared to 52.5% (*p* = 0.012) for the endoscopists. On sub‐group analysis for polyps ≤5 mm, the NPV for AI was significantly better at 90.9% on IEE compared to 55.0% for endoscopists (*p* = 0.024).

Similarly, the accuracy of AI for polyps ≤5 mm on IEE was superior to the endoscopists but despite a numerical trend, statistical significance was not achieved for polyps >5 mm. Table 3 demonstrates the comparison between the performance of CADx and endoscopists on IEE.

**TABLE 3:** Endoscopist versus artificial intelligence (AI) using image‐enhanced endoscopy

Polyp size	Evaluator	Sensitivity (95% CI)	*p*‐value	Specificity (95% CI)	*p*‐value	NPV (95% CI)	*p*‐value	Accuracy (95% CI)	*p*‐value
≤5 mm	AI	95.8 (87.5–100)	<0.001	66.7 (40.0–86.7)	0.436	90.9 (58.7–99.8)	0.024	84.6 (71.8–94.9)	<0.001
	Endoscopist	72.0 (62.5–81)		58.1 (48.6–66.7)		55.0 (44.7–65.0)		66.7 (59.7–73.3)	
>5 mm	AI	94.7 (84.2–100.0)	0.037	60.0 (20.0–100)	1.000	75.0 (19.4–99.4)	0.346	87.5 (70.8–100)	0.099
	Endoscopist	82.0 (73.7–90.2)		62.9 (37.1–88.6)		46.3 (30.7–62.6)		78.0 (68.5–86.3)	
Overall	AI	95.3 (88.4–100)	<0.001	65.0 (45.0–85.0	0.552	86.7 (59.5–98.3)	0.012	85.7 (76.2–93.7)	<0.001
	Endoscopist	76.4 (69.8–82.7)		59.3 (50.0–67.9)		52.5 (43.9–60.9)		71.0 (65.1–76.4)	

Abbreviations: AI, artificial intelligence; NPV, negative predicitve value.

### Performance of CADx for PIVI criteria

#### PIVI criterion for diagnose and leave hyperplastic diminutive polyps in the rectosigmoid region

On sub‐group analysis, we noted that the NPV for CADx for diminutive polyps in the colon was 80% on WL and 90.9% on IEE (Tables 1 and 2). CADx only met the PIVI criteria (90% or more) for using IEE.

#### PIVI criterion for the resect and discard diminutive adenomas in the colon

To determine if CADx fulfilled the ‘resect and discard’ strategy, we calculated the post‐polypectomy surveillance intervals based on CADx diagnosis of all diminutive colorectal polyps and histopathologic diagnosis of colorectal polyps >5 mm. This was then compared with the true surveillance intervals based on the histopathological diagnosis of all polyps (≤5 and >5 mm). We used both European Society of Gastrointestinal Endoscopy and British Society of Gastroenterology guidelines for calculating surveillance intervals.

Concordance between histology and CADx‐based surveillance intervals exceeded 90% on both WLI and IEE (Table 4).

**TABLE 4:** Concordance between histology and computer‐aided diagnosis (CADx)‐based surveillance intervals on white light and image‐enhanced endoscopy

**Agreement on surveillance intervals on WLI**		
BSG guidelines	39/43 patients	90.70%
ESGE guidelines	40/43 patients	93.02%
**Agreement on surveillance intervals on IEE**		
BSG guidelines	47/50 patients	94.00%
ESGE guidelines	48/50 patients	96.00%

Abbreviations: BSG, British Society of Gastroenterology; ESGE, European Society of Gastrointestinal Endoscopy; WLI, white light imaging.

## DISCUSSION

Our study provides external validation for a new CADx (WISE VISION; Japan) system and demonstrates that CADx performance can meet the PIVI thresholds for “resect and discard” of diminutive adenomas in the colon with both WLI and IEE. It can also meet the PIVI criterion for ‘diagnose and leave’ diminutive rectosigmoid hyperplastic polyps with IEE. We believe that this is the first report of a commercially available (CE marked) device that can work across all platforms and can meet PIVI thresholds on WLI as well as IEE like NBI and BLI.

We have also compared the performance of CADx with endoscopists with varied experience in optical diagnosis. Overall test sensitivity of the endoscopists was 76.4% which is similar to the reported sensitivity (76.1%) of endoscopists in previous studies.^15^

Real‐time optical diagnosis of polyps has the potential to transform clinical practice by reducing pathology & polypectomy costs, setting up surveillance intervals without waiting for histology, and reducing morbidity and procedure time. Histological analysis of just diminutive polyps alone costs one billion US dollars/year in the United States.^12^ It has been demonstrated that image enhancement technologies, such as NBI can meet the necessary performance parameters for a “resect and discard policy”. However, this requires specialist training, and attempts to achieve this outside of expert centers have been disappointing. It is hoped that with the advent of AI, this issue can be overcome. A recent systematic review and meta‐analysis suggested that AI models were consistently superior to non‐expert endoscopists in both the detection and characterization of polyps, including diminutive polyps.^25^ Our results are consistent with the findings of the above metanalysis which provides further evidence of the construct validity of our study.

Following is a case study comparing the optical diagnosis of that of an endoscopist and CADx.

**FIGURE 2:**
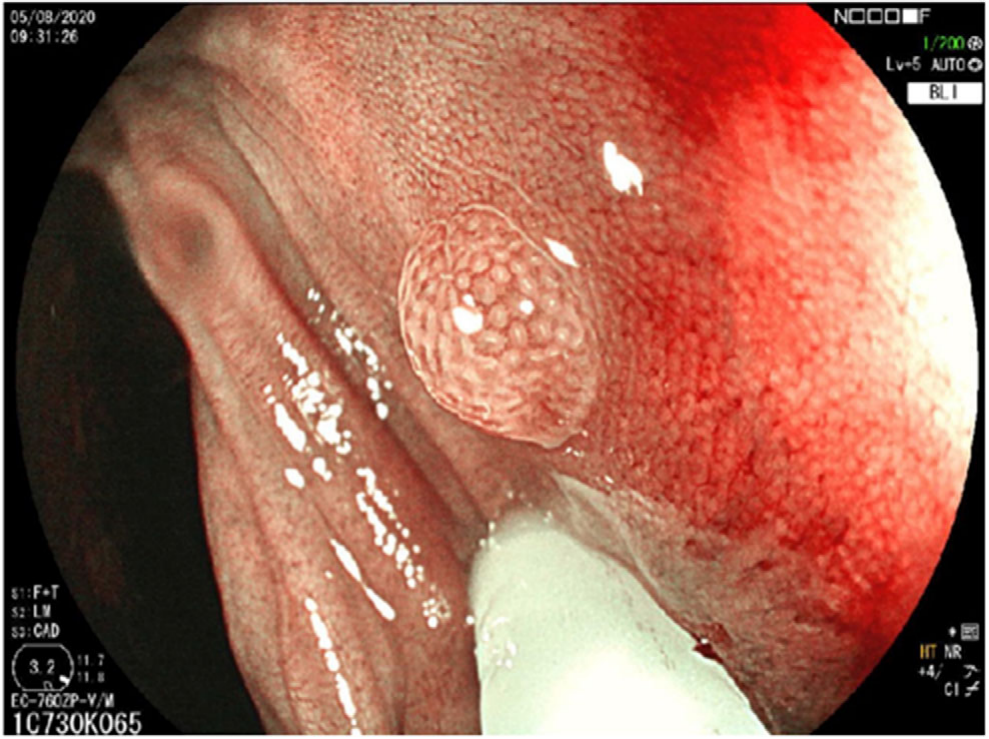
Case study: 2‐mm polyp noted in the sigmoid colon

A 2‐mm polyp (Figure 2) was noted in the sigmoid colon. The endoscopist reviewed the polyp on WLI as well as on IEE. On IEE, the endoscopist noted the polyp to have a smooth surface, round pits, and homogenous pit pattern but pericryptal vessels and therefore labeled the polyp as an adenoma, and hence removed it. The CADx system identified the above polyp as a hyperplastic polyp which was confirmed by histology.

Multiple groups have conducted studies in the field of AI application in the optical diagnosis of polyps. However, most of these studies are based on NBI. The initial CADx systems were made for NBI with near focus, which limited the widespread use of these systems.^20^, ^21^, ^22^, ^26^, ^27^ These systems also utilized a support vector machine (SVM), compared with more recent studies which use a more advanced Deep Neural Network (DNN) model. Integration of DNN into CADx has allowed systems to have higher diagnostic accuracy.

A recent study^23^ prospectively analyzed 267 images (133 on WLI and 134 on BLI) with endoscopists and CADx (Fujifilm Corp) where the accuracy of CADx was 84.2% compared to 75.2% for expert endoscopists on WLI and 83.6% compared to 79.3% for BLI. However, this study was limited to polyps examined by the Fujifilm Eluxeo colonoscopes. One of the big drawbacks of this study was that they didn't look at the sizes of polyps so couldn't express the results of diminutive polyps separately from the non‐diminutive polyps. The study did not report NPV nor did it look into surveillance intervals, so could not report on the PIVI criteria. The non‐experts only looked at the polyp images with CADx predicted diagnosis so again not possible to compare the performance of non‐experts with the CADx alone as reported by us. Byrne et al.^28^ published the performance of their convolutional neural network (CNN) model trained on the NICE classification of polyps, demonstrating an accuracy of 94% and NPV of 97%. However, their CNN model could only work on NBI and they did not perform a patient‐based analysis to report the PIVI figures. Zachariah et al.^29^ also developed a CNN base deep learning model where they achieved an accuracy of 94% for characterizing adenomas, and NPV of 97% for diminutive recto‐sigmoid polyps and a surveillance concordance of 93% between CADx and histology. Similar to our study, their CNN model worked on WL as well as NBI with comparable accuracy. However, they did not have any BLI images in their study which limits the generalization of their CNN model.

None of the published CADx studies have tested the ability of AI in white light and have also restricted the development of AI with single IEE technology like NBI or BLI. In our study, we have demonstrated the application of CADx with both white light and image enhancement using either NBI or BLI, thereby making it more universally applicable and offering a new paradigm independent of patented IEE technologies.

During subgroup analysis, we noticed that the performance of endoscopists is always better with polyps > 5 mm in size and also better with IEE as compared to WLI. This has been proven in all the previously published optical diagnosis studies with IEE and provides further proof of the construct validity of our study. However, an interesting fact is that the performance of AI was not dependent on size. AI also performed equally well on WLl and IEE on all parameters except NPV, which was better on IEE. We feel that future training of the model can address this minor discrepancy and make AI completely independent of size and type of light.

The strength of our study is that it is a well‐powered, planned trial comparing CADx with general endoscopists in which the CADx system performs significantly better than the endoscopists. We also demonstrate the applicability of the CADx system across different kinds of endoscopy platforms (Olympus and Fujifilm) on WLI, NBI, and BLI without any magnifications.

We strongly believe that the future CADx systems should not just simulate the conventional human practice but should go a step further and make an optical diagnosis on white light without the need for IEE, dye sprays, magnifications, or need for special endoscopes. We have shown that CADx with WLI meets the PIVI threshold for resect and discard strategy and fell just short of meeting the threshold for diagnose and leave. A slightly enhanced optimization can enable it to meet the latter threshold on WLI as well.

The limitations of our study are that it is an image‐based study, although images were extracted from real‐time videos. However, it needs to be tested in real time during the colonoscopy. Furthermore, the number of polyps with NBI and BLI were not equally distributed so cross‐comparison or subgroup analysis could not be performed.

Our data gives us confidence that this CADx system is ready to be used in real time

in‐vivo diagnostic trials and if the performance can be replicated during real time use then it raises real hope for the introduction of resect and discard as well as diagnose and leave strategy in clinical practice.

## CONCLUSION

Real‐time polyp characterization has been challenging for endoscopists and while possible is generally only applied in specialist settings. Our data suggest that AI‐based optical diagnosis is promising and has the potential to be significantly better than the performance of general endoscopists. We believe that AI can help make real‐time optical diagnoses of polyps meeting the PIVI standards set by the American Society of Gastrointestinal Endoscopy and paving way for the widespread adoption of the ‘resect and discard’ as well as ‘diagnose and leave’ strategy in clinical practice.

## CONFLICT OF INTEREST

None.

## FUNDING INFORMATION

None.

## References

[1] Hill MJ , Morson BC , Bussey HJR . Aetiology of adenoma–carcinoma sequence in large bowel. Lancet 1978; 311: 245–7. 10.1016/s0140-6736(78)90487-7 74668

[2] Winawer SJ , Zauber AG , Ho MN *et al*. Prevention of colorectal cancer by colonoscopic polypectomy. N Engl J Med 1993; 329: 1977–81. 8247072 10.1056/NEJM199312303292701

[3] Kaminski MF , Regula J , Kraszewska E *et al*. Quality indicators for colonoscopy and the risk of interval cancer. N Engl J Med 2010; 362: 1795–803. 20463339 10.1056/NEJMoa0907667

[4] Kaminski MF , Wieszczy P , Rupinski M *et al*. Increased rate of adenoma detection associated with reduced risk of colorectal cancer and death. Gastroenterology 2017; 153: 98–105. 28428142 10.1053/j.gastro.2017.04.006

[5] Butterly LF , Chase MP , Pohl H *et al*. Prevalence of clinically important histology in small adenomas. Clin Gastroenterol Hepatol 2006; 4: 343–8 16527698 10.1016/j.cgh.2005.12.021

[6] Yoo TW , Park DI , Kim YH *et al*. Clinical significance of small colorectal adenoma less than 10 mm: The KASID study. Hepatogastroenterology 2007; 54: 418–21. 17523287

[7] Ponugoti PL , Cummings OW , Rex DK . Risk of cancer in small and diminutive colorectal polyps. Dig Liver Dis 2017; 49: 34–7. 27443490 10.1016/j.dld.2016.06.025

[8] Gupta N , Bansal A , Rao D *et al*. Prevalence of advanced histological features in diminutive and small colon polyps. Gastrointest Endosc 2012; 75: 1022–30. 22405698 10.1016/j.gie.2012.01.020

[9] Denis B , Bottlaender J , Weiss AM *et al*. Some diminutive colorectal polyps can be removed and discarded without pathologic examination. Endoscopy 2011; 43: 81–6. 21108174 10.1055/s-0030-1255952

[10] Tsai FC , Strum WB . Prevalence of advanced adenomas in small and diminutive colon polyps using direct measurement of size. Dig Dis Sci 2011; 56: 2384–8. 21318587 10.1007/s10620-011-1598-x

[11] Chaput U , Alberto SF , Terris B *et al*. Risk factors for advanced adenomas amongst small and diminutive colorectal polyps: A prospective monocenter study. Dig Liver Dis 2011; 43: 609–12. 21764012 10.1016/j.dld.2011.02.002

[12] Abu Dayyeh BK , Thosani N , Konda V *et al*. ASGE technology committee systematic review and meta‐analysis assessing the ASGE PIVI thresholds for adopting real‐time endoscopic assessment of the histology of diminutive colorectal polyps. Gastrointest Endosc 2015; 81: 502–16. 25597420 10.1016/j.gie.2014.12.022

[13] Rex DK , Kahi C , O'Brien M *et al*. The American Society for Gastrointestinal Endoscopy PIVI (Preservation and Incorporation of Valuable Endoscopic Innovations) on real‐time endoscopic assessment of the histology of diminutive colorectal polyps. Gastrointest Endosc 2011; 73: 419–22. 21353837 10.1016/j.gie.2011.01.023

[14] Houwen B , Hassan C , Coupe V *et al*. Definition of competence standards for optical diagnosis of diminutive colorectal polyps: European Society of Gastrointestinal Endoscopy (ESGE) position statement. Endoscopy 2022; 54; 88–99 34872120 10.1055/a-1689-5130

[15] Rees C , Rajashekar P , Wilson A *et al*. Narrow band imaging optical diagnosis of small colorectal polyps in routine clinical practice: The detect inspect characterise resect and discard 2 (DISCARD 2) study. Gut 2017; 66: 887–95. 27196576 10.1136/gutjnl-2015-310584 PMC5531217

[16] Neumann H , Vieth M , Bisscops R *et al*. Leaving colorectal polyps in place can be achieved with high accuracy using blue light imaging (BLI). United European Gastroenterol J 2018; 6: 1099–105. 10.1177/2050640618769731 PMC6137600 30228899

[17] Rondonotti E , Paggi S , Amato A *et al*. Blue‐light imaging compared with high‐definition white light for real‐time histology prediction of colorectal polyps less than 1 centimeter: A prospective randomized study. Gastrointest Endosc 2019; 89: 554–64.e1. 30273590 10.1016/j.gie.2018.09.027

[18] Neumann H , Fujishiro M , Wilcox C *et al*. Present and future perspectives of virtual chromoendoscopy with i‐scan and optical enhancement technology. Dig Endosc 2013; 26(Suppl 1): 43–51. 10.1111/den.12190 24373000

[19] Vleugels JLA , Hazewinkel Y , Dijkgraa MGW . Optical diagnosis expanded to small polyps: Post‐hoc analysis of diagnostic performance in a prospective multicenter study. Endoscopy 2019; 51: 244–52. 30544284 10.1055/a-0759-1605

[20] Mori Y , Kudo SE , Misawa M . *et al*. Real‐time use of artificial intelligence in identification of diminutive polyps during colonoscopy: A prospective study. Ann Intern Med 2018; 169: 357–66. 30105375 10.7326/M18-0249

[21] Gross, S , Trautwein, C , Behrens, A *et al*. Computer‐based classification of small colorectal polyps by using narrow‐band imaging with optical magnification. Gastrointest Endosc 2011; 74: 1354–9 22000791 10.1016/j.gie.2011.08.001

[22] Kominami Y , Yoshida S , Tanaka S *et al*. Computer‐aided diagnosis of colorectal polyp histology by using a real‐time image recognition system and narrow‐band imaging magnifying colonoscopy. Gastrointest Endosc 2016; 83: 643–9. 26264431 10.1016/j.gie.2015.08.004

[23] Weigt J , Repici A , Antonelli G *et al*. Performance of a new integrated computer‐assisted system (CADe/CADx) for detection and characterization of colorectal neoplasia. Endoscopy 2022; 54: 180–4. 33494106 10.1055/a-1372-0419

[24] https://www.businesswire.com/news/home/20210111006087/en/.

[25] Lui TKL , Guo CG , Leung WK . Accuracy of artificial intelligence on histology prediction and detection of colorectal polyps: A systematic review and meta‐analysis. Gastrointest Endosc 2020; 92: 11–22.e6. 32119938 10.1016/j.gie.2020.02.033

[26] Takemura Y , Yoshida S , Tanaka S *et al*. Computer‐aided system for predicting the histology of colorectal tumors by using narrow‐band imaging magnifying colonoscopy. Gastrointest Endosc 2012; 75: 179–85 22196816 10.1016/j.gie.2011.08.051

[27] Misawa M , Kudo SE , Mori Y *et al*. Accuracy of computer‐aided diagnosis based on narrow‐band imaging endocytoscopy for diagnosing colorectal lesions: Comparison with experts. Int J Comput Assist Radiol Surg 2017; 12: 757–66. 28247214 10.1007/s11548-017-1542-4

[28] Byrne MF , Chapados N , Soudan F *et al.* Real‐time differentiation of adenomatous and hyperplastic diminutive colorectal polyps during analysis of unaltered videos of standard colonoscopy using a deep learning model. Gut 2019; 68: 94–100. 29066576 10.1136/gutjnl-2017-314547 PMC6839831

[29] Zachariah R , Samarasena J , Luba D *et al*. Prediction of polyp pathology using convolutional neural networks achieves “resect and discard” thresholds. Am J Gastroenterol 2020; 115: 138–44 31651444 10.14309/ajg.0000000000000429 PMC6940529

